# Application of Physiologically Based Pharmacokinetic Models in Chemical Risk Assessment

**DOI:** 10.1155/2012/904603

**Published:** 2012-03-19

**Authors:** Moiz Mumtaz, Jeffrey Fisher, Benjamin Blount, Patricia Ruiz

**Affiliations:** ^1^Computational Toxicology and Methods Development Laboratory, Division of Toxicology and Environmental Medicine (DTEM), Agency for Toxic Substances and Disease Registry (ATSDR), Atlanta, GA 30333, USA; ^2^National Center for Toxicological Research, USFDA, Jefferson, AR 72079, USA; ^3^Division of Laboratory Studies, National Center for Environmental Health, Centers for Disease Control and Prevention (CDC), Atlanta, GA 30341, USA

## Abstract

Post-exposure risk assessment of chemical and environmental stressors is a public health challenge. Linking exposure to health outcomes is a 4-step process: exposure assessment, hazard identification, dose response assessment, and risk characterization. This process is increasingly adopting “in silico” tools such as physiologically based pharmacokinetic (PBPK) models to fine-tune exposure assessments and determine internal doses in target organs/tissues. Many excellent PBPK models have been developed. But most, because of their scientific sophistication, have found limited field application—health assessors rarely use them. Over the years, government agencies, stakeholders/partners, and the scientific community have attempted to use these models or their underlying principles in combination with other practical procedures. During the past two decades, through cooperative agreements and contracts at several research and higher education institutions, ATSDR funded translational research has encouraged the use of various types of models. Such collaborative efforts have led to the development and use of transparent and user-friendly models. The “human PBPK model toolkit” is one such project. While not necessarily state of the art, this toolkit is sufficiently accurate for screening purposes. Highlighted in this paper are some selected examples of environmental and occupational exposure assessments of chemicals and their mixtures.

## 1. Background

As industrial society inhabitants, we are exposed to hundreds of chemicals and to an increasing number of chemical combinations, as mixtures. Exposure to multiple chemicals simultaneously or sequentially is the rule rather than the exception [1]. Chemical risk assessments estimate public health consequences from exposure—specifically, exposure to environmental, occupational, or therapeutic chemicals. Most often, estimates of unintentional exposures are based on imprecise metrics of external (air, water, and soil) concentrations and default exposure factors. Chemical exposure assessment thus continues to challenge public health and environmental protection.

**Figure 1:**
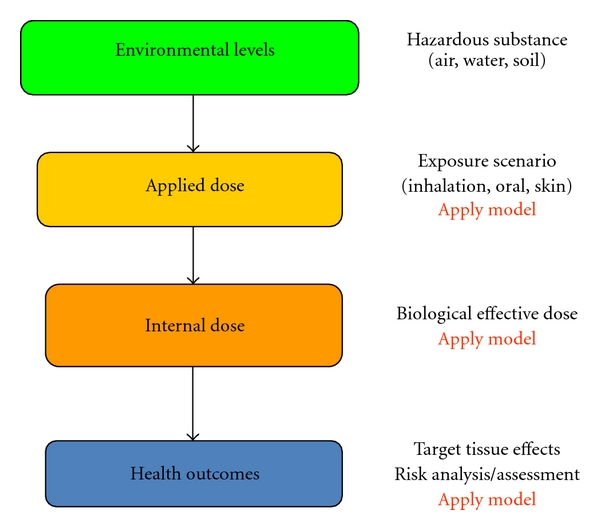
Application of in silico models in establishing the cause and effect relationship.

Evaluation of human exposure data in the context of public health (i.e., the linking of exposures to health outcome through the establishment of the cause-effect relationship) is a complex process. To establish this relationship, several traditional programs have been used, including health surveillance and disease registries. Through these programs, researchers closely monitor chemical releases in the environment and conduct health studies. When exposed cohorts of human populations are identified, disease registries are established. The cause-effect relationship brings together several biochemical and molecular processes such as nature of the exposures, pharmacokinetics/pharmacodynamics of the chemical(s), and interactions of the biologically effective moiety with the target tissue macromolecules [2]. Critical data gaps exist in these cause-effect relationships. In the absence of a complete set of data, in silico/computational tools can help fill the data gaps [3–8] (Figure 1). In silico modeling employs the mathematical know-how and the computer science advances to evaluate exposures and sometimes to predict risk posed by chemicals.

Several in silico models are used to track the movement of chemicals through the environment and through the human body. Physiologically based pharmacokinetic (PBPK) models are a family of such tools; their potential applications in human health risk assessment have stirred considerable interest. The salient feature of PBPK models is that through simulation they can approximate the kinetic behavior of chemical(s). The models are actually designed to integrate the physical and biological characteristics of a chemical with the physiological happenings in the body to estimate internal dose in target tissues/organs.

In 1995, a group of experts were brought together by ATSDR to advice on the application of computational tools for human risk assessment of toxic substances [9]. Since then, in silico modeling has been applied, where possible, for hazard identification and to determine internal dose through quantitative structure-activity relationship (QSAR), PBPK, PBPK/pharmacodynamic, and biologically based dose-response (BBDR) modeling [10–15].

Engaged academics, stakeholders, and other interested parties have supported modeling projects, advancement of model development, and model applications. We have highlighted in this paper selected PBPK model applications at the Agency for Toxic Substances and Disease Registry (ATSDR) and National Center for Environmental Health (NCEH), Centers for Disease Control and Prevention (CDC), that led to derivation of minimal risk levels (MRLs); risk assessment of mixtures; assessment of occupational exposures; site specific assessment; interpretation of human biomonitoring data.

## 2. PBPK Modeling and Minimal Risk Levels (MRLs)

Minimal risk levels (MRLs) are estimates of daily human exposure to a hazardous substance at or below which that substance is unlikely to pose a measurable risk of harmful (adverse), noncancerous effects. MRLs are calculated for an exposure route (inhalation or oral) over a specified period (acute, intermediate, or chronic). MRL calculations are typically based on toxicity studies conducted with specific routes of administration. From such data no observed adverse effect levels (NOAELs) or lowest observed adverse effect levels (LOAELs) are identified and adjusted for associated uncertainties. Sometimes data might be appropriate for MRL derivation from one route but not from another. Database gaps or lack of suitable experimental studies prevent using this empirical approach to derive MRLs for all priority pollutants and for all routes of potential exposure. To circumvent this problem, we examined the potential use of PBPK modeling by studying methylene chloride (MC) and trichloroethylene (TCE). PBPK models were available, and MRLs were derived for both these chemicals of interest [16–19]. We used PBPK models to derive each MRL using the study that had provided the basis for the then-current MRL.

In general, the MRLs derived using PBPK models were somewhat higher compared with those derived using the traditional approach. For MC, PBPK-derived MRLs tended to be higher (ratio of 2 to 15) for both inhalation and oral exposure. For TCE, PBPK-derived MRLs tended to be slightly higher (ratio of 1 to 4) for inhalation exposure but yielded much lower MRLs (ratio of 0.03 to 0.20) for oral exposure. These differences were chemical, exposure route-dependent, and varied as a function of dose metric used. The general conclusion of this exercise was that a full PBPK model may not be necessary to derive an MRL—a good understanding and application of basic pharmacokinetic principles might suffice [16].

## 3. PBPK/PD Modeling of Mixtures

Exposure to multiple chemical or nonchemical stressors is a fact of life. Recently, more attention has been paid to the risk assessment of such exposures [1]. Because PBPK models can incorporate pharmacodynamic characteristics of a chemical, they can be employed in cumulative risk assessment for exposure to multiple chemicals [20]. As methods of chemical mixture risk assessment have evolved during the past two decades, they have undergone drastic changes. Initially, single chemical methods, with slight modifications, were used to evaluate simple mixture toxicities. Then, specific methods were developed to advance these methods further. These, in turn, were followed by the first generation of PBPK modeling approaches. Now, they are being replaced by the more advanced second-generation PBPK/PD models. From the first generation's evaluation of simple mixtures to the second generation's evaluation of more complex mixtures, the development process has gone through different phases. Today's sophisticated models allow integration of concurrent exposure to multiple chemicals through integrating cellular and molecular biology information of the component chemicals and available mechanistic data [21].

If more than one chemical enters the body, a potential arises for interactions among chemicals, their metabolites, and the biological molecules/systems. Interaction threshold (IT) is defined as combined total dose of chemicals at which interactions become significant in terms of joint toxicity of a mixture. In most cases, experimental determination of low-dose interaction thresholds is economically infeasible—it needs a large number of laboratory animals. Instead, researchers have used an empirical weight-of-evidence methodology to evaluate binary interactions [22]. This methodology incorporates a “bottom-up” approach to evaluate multicomponent mixtures [22, 23].

If, however, appropriate data are available, PBPK/PD modeling actually is better than any other method to simulate various exposure scenarios and to study interactions. These models can also address episodic or pulse exposures. We studied interactions between two organophosphates: chlorpyrifos and parathion [24, 25]. They are both potent pesticides found together in the environment, have similar metabolic pathways, and the parent chemicals, and their respective metabolites cause toxicity by inhibition of acetylcholinesterase (AchE). Chlorpyrifos is rapidly desulfurated by CYP450 3A4 and 2D6 to chlorpyrifos-oxon. Chlorpyrifos-oxon is 300 to 400 times more potent at inhibiting rat brain AChE than is chlorpyrifos. Parathion is desulfurated in the liver by CYP450 3A4, 3A5, 1A2, and 2D6 to paraoxon. Paraoxon is also a much more active inhibitor of AChE than is its parent. The same isoenzymes—P450, 3A4, and 2D6—are involved in the metabolism of both chemicals to the oxon that inhibits AChE.

**Figure 2:**
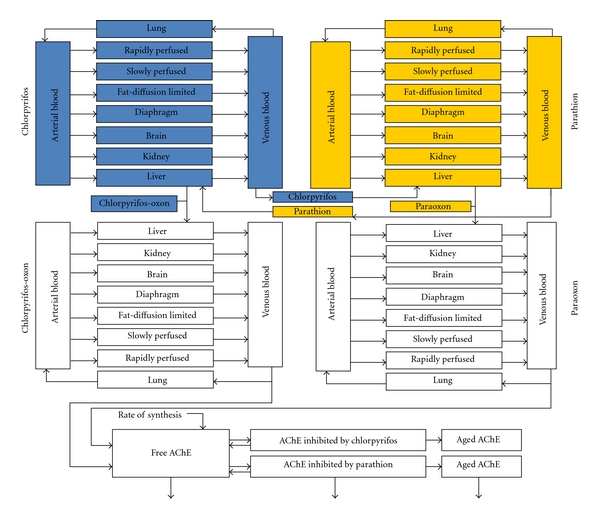
A schematic diagram of the physiologically based pharmacokinetic/pharmacodynamic (PBPK/PD) model of acetylcholinesterase (AchE) inhibition by binary mixtures of chlorpyrifos, parathion, and their metabolites: chlorpyrifos-oxon and paraoxon.

Thus, to evaluate the PK and PD interactions between chlorpyrifos and parathion, we developed a mixture PBPK/PD model that comprised four individual submodels (chlorpyrifos, chlorpyrifos-oxon, parathion, paraoxon) [25]. The two parent models were linked to their metabolite models through the liver compartments (Figure 2). The predicted metabolite concentrations in blood were linked to a PD model for AChE kinetics, where the competition for cholinesterase occurs. Model simulations indicated that for each chemical, additivity takes place at oral dose levels below 0.08 mg/kg. At higher doses, antagonism by enzymatic competitive inhibition occurs. We determined the interaction threshold by comparing the levels of simulated mixture response with levels anticipated from the individual response addition. In this modeling exercise, we showed that PBPK/PD modeling can improve experimental study design and can help risk assessors to quantify mixture risks for low-dose exposures.

Still, for a mixture that contains a large number of chemicals, a “bottom-up” approach could be tedious and impractical. Petroleum hydrocarbon mixtures such as gasoline, diesel fuel, aviation fuel, and asphalt liquids typically contain hundreds of compounds. These compounds include aliphatic and aromatic hydrocarbons within a specific molecular weight range and sometimes lesser amounts of additives. And they often exhibit qualitatively similar pharmacokinetic (PK) and pharmacodynamic (PD) properties. Nevertheless, some components do exhibit specific biological effects, such as methyl *t*-butyl ether and benzene in gasoline. One of the potential pharmacokinetic interactions of many components in such mixtures is inhibition of the metabolism of some components. Due to the mixtures' complexity, a quantitative description of the pharmacokinetics of each component has not been available, particularly in the context of differing blends of these mixtures.

Consequently, we developed a PBPK modeling approach to describe the kinetics of whole gasoline [26]. The approach simplifies the problem by isolating specific components for which a description is helpful and by treating the remaining components as a single, lumped chemical. In this manner, the effect of the nonisolated components (i.e., inhibition) is taken into account. As previously shown for simple mixtures, this gasoline model was developed by linking at the binary level individual PBPK models through the liver compartment—where competitive inhibition of hepatic metabolism occurs. During gas-uptake kinetics experiments in rats exposed to whole gasoline, kinetics data were used for the single chemicals, for simple mixtures of the isolated chemicals, and for the isolated and lumped chemicals. While some sacrifice in model accuracy occurs with a chemical-lumping approach, such a model still affords a good representation of the kinetics of five isolated chemicals (*n*-hexane, benzene, toluene, ethylbenzene, and *o*-xylene) during exposure to various levels of two different gasoline blends. When appropriate kinetics data are available for model development, the approach could be applicable to other hydrocarbon mixtures.

## 4. A Biologically Based Dose-Response (BBDR) Model of Hypothalamic-Pituitary-Thyroid Axis

Some environmental chemicals affect endocrine function. Even at very low levels, these chemicals could alter hormone systems. The hypothalamic-pituitary-thyroid (HPT) axis controls many physiologic functions, including metabolism, development, and reproduction. A biologically based dose-response (BBDR) model for adult rats includes submodels for dietary iodide, thyroid-stimulating hormone (TSH), and the thyroid hormones thyroxine (T4) and 3,5,3′- triiodothyronine (T3) [27, 28]. The independently developed individual submodels were linked together to form the endogeneous BBDR-HPT axis model. The resultant model incorporates key biological processes, including

1. the influence of T4 on TSH production (the HPT axis negative feedback loop),
2. stimulation of thyroidal T4 and T3 production by TSH,
3. TSH upregulation of the thyroid sodium (Na(+))/iodide symporter,
4. recycling of iodide from metabolism of thyroid hormones.

The model was calibrated to predict steady-state concentrations of iodide, T4, T3, and TSH for the euthyroid rat, whose dietary intake of iodide was 20 *μ*g/day. Then, the model was used to predict perturbations in the HPT axis caused by insufficient dietary iodide intake. Simulation results were compared with experimental findings. The BBDR-HPT axis model was successful in simulating perturbations in serum T4, TSH, and thyroid iodide stores for low-iodide diets. When dietary iodide intake becomes insufficient to sustain the HPT axis, the model simulations show a steep dose-response relationship between dietary iodide intake and serum T4 and TSH. This BBDR-HPT axis model might link with PBPK models for thyroid-active chemicals to evaluate and predict dose-dependent HPT axis alterations based on hypothesized modes of action.

## 5. PBPK Modeling in Occupational Exposures Studies

In another study, we examined the potential toxicity from coexposure to three CNS depressants: toluene, ethylbenzene, and xylene were evaluated under resting and working conditions [29]. Under OSHA and American Conference of Governmental Industrial Hygienists (ACGIH) guidelines, the mixture formula (unity calculation) provides a method for evaluating exposures to mixtures of chemicals that cause similar toxicities [30]. According to the formula, if exposures are reduced in proportion to the number of chemicals and their respective exposure limits, the overall exposure is acceptable. Most of the occupational exposure limits are derived from studies of resting humans or animals. But this approach assumes that responses are additive. To determine the additivity assumption's validity, unity calculations were performed for a variety of exposures to toluene, to ethylbenzene, to xylene, or to all three. In the calculation, the concentration of each chemical in blood was used rather than the inhaled concentration. The blood concentrations were predicted using a validated PBPK model to allow exploration of a variety of exposure scenarios. At rest, a modest overexposure—2.9 to 4.6 times—occurs due to pharmacokinetic interactions. But the study showed that workers with higher activity might experience a significantly higher absorbed dose that could result in 87% higher internal doses. This study showed the importance of work load's effect on internal chemical doses.

## 6. Site-Specific Assessment Integrating PBPK/QSAR Modeling

In a highly contaminated residential area, total polychlorinated biphenyls (PCBs) soil levels ranged from 17.4 to 840 mg/kg—much higher than the maximum soil level of 1.5 mg/kg reported nationally [31]. The national average range of PCBs in serum is 4–7 *μ*g/L, but the serum levels of some of its residents ranged between 76.3 and 187.5 *μ*g/L [10]. The major human exposure to PCBs is through ingestion of contaminated food. We wanted to determine whether soil ingestion contributed to these higher levels in the serum of area residents. PBPK models of the 25 most common PCB congeners were developed based on a published method [32]. Partition coefficients and metabolic constants for the models were determined using published QSAR procedures [33, 34].

**Figure 3:**
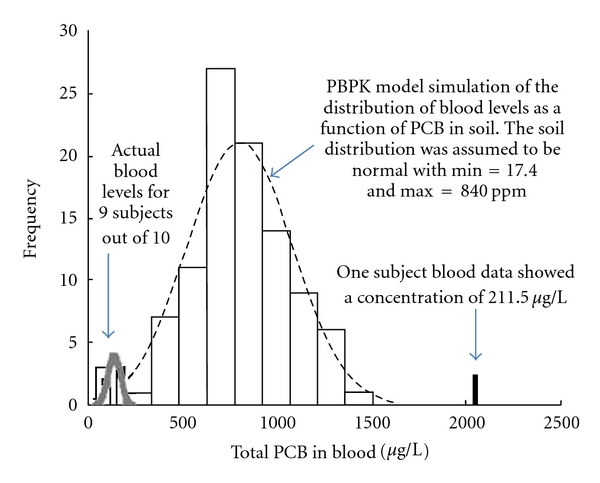
The distribution of actual levels of total PCBs in blood near a waste site.

The models were then used to estimate the contribution of these 25 PCB congeners through soil ingestion to the levels of serum PCBs. PBPK simulations were run using a soil ingestion default rate of 50 mg/day for a lifetime exposure scenario. Simulations using average national soil levels showed that only 0.6% of the total PCBs levels are from soil. Thus, nationwide soil ingestion was not a major contributor to serum blood levels. To confirm this in the contaminated area residents, a probabilistic distribution for PCB blood levels was derived based on the actual PCB soil measurements of the area. The distribution was then applied to the 25 PBPK models to derive a distribution of predicted total PCBs in blood for lifetime exposure scenarios. The derived distribution of blood levels was superimposed on the actual distribution of measured serum levels. The distribution of actual blood levels for 9 out of 10 persons fell within the modeled exposure range, while the mean of the actual blood levels distribution fell within the 2-percentile, lower end of the simulated curve (Figure 3).

Thus, in this community, soil did not appear to contribute significantly to serum levels. Because of lack of actual exposure data of the community, however, the simulations were run on default exposure assumptions. Dietary intake (e.g., fish) or inhalation could be the alternative source of PCBs serum levels. These types of PBPK modeling studies help us determine relative contribution of environmental media to the overall internal doses of chemicals.

## 7. Human PBPK Tool Kit Development: The General Approach

To better serve health assessors and increase their use, we are developing a “human PBPK model toolkit” to assist with site-specific health assessments [35–37]. This toolkit will comprise a series of published models coded in Berkeley Madonna—a common simulation language [36]. Ultimately a Web linkage to a PBPK database will be available where health assessors and other related health workers can access easily many different models for use in assessment activities.

At the outset, we conducted literature review to identify available human PBPK models for the chemicals of interest. Following literature searches of human health-related databases such as Medline, Toxline, and PubMed, we identified hundreds of PBPK models. These models varied in their complexity based on the scientific understanding of the chemistry, biological behavior, and insights gained into the mechanism(s) or mode of action of a given chemical's toxicity. Thus, the models contained different numbers of compartments (e.g., liver, kidney, and other organs). Often the compartments were designed for parent chemicals, but some included metabolite(s). The criteria we used for model selection included critical scientific issues such as the number of datasets used to calibrate and evaluate the model, the model's maturity (number of predecessor models from which the model was derived), and the author's experience. Currently, the toolkit includes models that are at various stages of development for environmental contaminants, including volatile organic compounds (VOCs) and metals [12, 13, 37].

We also developed a generic 7-compartment VOC model; it consists of blood, fat, skin, kidney, and liver, rapidly and slowly perfused tissue compartments, and a gas exchange compartment [37]. We included these compartments in the model based on their use in previously published PBPK models [38–43]. The generic VOC PBPK model can be used for six VOCs:

- benzene (BEN),
- carbon tetrachloride (CCl_4_),
- dichloromethane (DCM),
- perchloroethylene (PCE),
- trichloroethylene (TCE),
- vinyl chloride (VC).

All compartments were described as well mixed and flow limited. We obtained chemical-specific and biochemical parameters for the model from published literature [41–48]. The model code allowed simulation of three routes of exposure, either individually or simultaneously: inhalation, oral ingestion, and dermal absorption. Due to the lack of available published human datasets, we did not conduct a comparison of the generic model predictions for dermal route. In the current model version, we also did not include original-model simulations for metabolites and metabolite data. Nevertheless, a critical future improvement for this model's postscreening use is incorporation of metabolite information, particularly when metabolite(s) mediate toxicity.

Our first test of the model's applicability was to compare the published human kinetic data for each VOC with the corresponding published model predictions. To test further the model's reliability, we calculated the area under the concentration curve (AUC) for blood or exhaled breath for each VOC using both our generic and original model. For each kinetic time course dataset, we also calculated the mean of the sum of the squared differences (MSSDs) between model prediction and observation. MSSD was computed by squaring the difference between a measured data point and the value of the simulation at the corresponding time. Then, the summed squares were divided by the number of data points. The MSSD was thus determined for both the published model and for our generic VOCs model [37].

For each of the specific VOCs, we used the VOC PBPK model to estimate the blood concentrations for the available minimal risk levels (MRLs) values [49–54]. We repeated this process for each VOC for which biomonitoring data on human blood levels were available from the National Health and Nutrition Examination Survey (NHANES) [55]. Steady-state VOC concentrations in venous blood were then compared with NHANES data using these simplified assumptions about exposure frequency and duration. If the measured NHANES blood levels were below those estimated from the simulations, the exposures would be regarded as “safe” [37].

We also reviewed published human metals PBPK models for arsenic, mercury, and cadmium [56–61]. We selected the best model available based on performance, accuracy, and reproducibility and recoded them using Berkeley Madonna [36]. We took from the literature human physiological and chemical-specific parameters describing the absorption, distribution, and blood and tissue partitioning of As, Hg, and Cd. The PBPK models allow simulation of different routes of exposure, either individually or simultaneously.

A published Cd toxicokinetic model [59–61] describes aggregated lung, liver, kidney, blood, and other tissues. Intake by oral and inhalation routes are transferred to an uptake pool that distributes to three blood compartments [61]. The model predicted the urinary concentrations of Cd considered a surrogate for body burden in assessing health risk from exposure, including the sex- and age-stratified geometric urinary mean. This model was used to predict the creatinine-corrected urinary Cd concentrations among females and males from the *Fourth National Report on Human Exposure to Environmental Chemicals* [55].

We recoded a human PBPK model for arsenic. It consists of interconnected submodels for inorganic arsenic and its metabolites, monomethyl arsenic (MMA), and dimethylarsenic (DMA) [56]. It includes compartments for lung, liver, GI tract, kidney, muscle, brain, skin, and the heart. Single or continuous oral exposures to inorganic arsenic in the +3 or +5 valence state or exposures via drinking water were simulated. The recoded model adequately simulated experimental human data found in the published literature [56]. Using a visual comparison, the model performance was in good agreement with the original model. We evaluated performance by calculating values for percent median absolute performance error (MAPE %), median performance error (MPE %), and root median square performance error (RMSPE %) based on estimates of performance error (PE) [12].

We recoded a human toxicokinetic model for methylmercury based on the Carrier et al. model [12, 57, 58]. The model consists of a total body compartment. By a first-order process, methylmercury enters this compartment from the GI tract. The amount of methylmercury in blood is proportional to that in the total body compartment. The recoded model reproduced all the simulations of the original model [57, 58]. By visual comparison, the model performance was in good agreement with the original model. We evaluated the model performance by calculating a value for percent median absolute performance error (MAPE %), median performance error (MPE %), and root median square performance error (RMSPE %) based on estimates-of-performance error (PE). The model could simulate and could accurately predict the available total body burden of mercury experimental data. The model predictions were similar to those observed experimentally and found in published literature. Overall, the current model could integrate those various experimental data that are critical determinants of methylmercury kinetics. The current model duplicates the time courses of various tissue burdens for different dose regimens and exposure scenarios.

## 8. PBPK: Biomonitoring Data and Its Interpretation

Several population-representative biomonitoring programs are underway in Canada, California, Asia, and Europe. These biomonitoring programs are similar to CDC's National Health and Nutrition Examination Survey (NHANES) that provides representative data for the United States. For risk managers, the growing availability of such data for hundreds of chemicals provides an opportunity as well as a challenge. To address the interpretation of such data, several alternative methods have been proposed, such as reverse dosimetry [62] and biomonitoring equivalents (BEs) [63].

The reverse dosimetry approach employs PBPK models as a tool; this tool interprets NHANES data to estimate the intake dose or external environmental concentration based on a measured tissue concentration [64–68]. We used the chloroform PBPK model [48] in combination with a mass transfer model [69] that describes the transfer of volatile organic chemicals (VOCs) from water to air during showering—an event that contributes significantly to VOC inhalation exposure. We incorporated exposure contributions from multiroutes and -sources into the published PBPK model [48]. This integrated model was used to predict chloroform concentrations in blood and exhaled breath from multiroute exposure to chloroform in the general population. MATLAB Simulink, the graphical simulation tool (The Math-Works, Inc., Natick, MA) was used for time-course and dose-response simulations, with a Monte Carlo sensitivity analysis. The predictive ability of this combined model was evaluated with three published studies that provided exhaled breath or blood chloroform concentrations. The studies also gave the most complete descriptions of how the volunteers were exposed and when the exhaled breath or blood samples were collected. To make it as close to reality as possible, we varied—together with other parameters in the model—the time of blood and exhaled breath samples collection and starting time of showering and water drinking. We ran the model for 10,000 iterations.

Reverse dosimetry was carried out by performing Monte Carlo analysis using appropriate varied timing of sampling and exposure. A reference chloroform concentration in water (1 *μ*g/L) was then used to predict the distribution of chloroform concentrations in blood (pg/mL). The values thus obtained were then inverted to obtain a distribution of an “exposure conversion factor” (ECF) in (*μ*g/L in water)/(pg/mL in blood). The distribution of the ECF can be multiplied by any observed chloroform concentrations in blood to estimate a distribution of chloroform concentrations in water to which a person might have been exposed. Our original assumption was that a simple structured PBPK model was adequate—a complex model increased the number of parameters and associated uncertainties. On the contrary, we found that a comprehensive exposure regimen was needed to aggregate all major contributing factors, including spatial and temporal profiles of chloroform in water, chloroform in ambient air, human activities, and water consumption patterns.

Showering was shown to yield much higher chloroform concentrations in blood than did water drinking—a conclusion consistent with previous experimental study. Still, that chloroform *metabolites* induce cytolethality in target tissues but not the parent compound is well known. Despite chloroform's higher concentration in blood after showering than after water-drinking exposure, that more chloroform is metabolized (first-pass) after water drinking exposure is possible and may exert more toxicity compared with showering exposure. This experience shows that only by integrating biomonitoring and PBPK/PD modeling techniques into both exposure and risk assessments can we obtain a more scientific basis for regulatory decisions that protect the public health.

Biomonitoring equivalents (BEs) are also used to interpret exposures [63, 70–72]. BEs estimate the concentration of a chemical or its metabolite in a biological medium consistent with an existing exposure guidance value such as a tolerable daily intake, minimal risk levels, or reference dose. The BE approach integrates available pharmacokinetic data necessary to convert, in a biological medium, a current exposure guidance value into an equivalent concentration. A range of pharmacokinetic data and approaches not limited to PBPK modeling is used to derive BE values. If human pharmacokinetic information is available, a target external dose is converted into the corresponding expected internal dose (concentration of parent compound or metabolite in blood or urine or both) in humans. Alternatively, if pharmacokinetic data are available in the animal species used in the study, those data provide the point of departure (POD) on which the exposure guidance value is based. The internal dose is then estimated in the animal at the POD. The appropriate uncertainty factors (UFs) then correspond to those used in the derivation of the exposure guidance value and are applied to derive a BE.

We also collected risk assessment-based chronic exposure reference values such as reference doses (RfDs), reference concentrations (RfCs), tolerable daily intakes (TDIs), cancer slope factors, and key pharmacokinetic model parameters for 47 VOCs [71]. Using steady-state solutions to a generic PBPK model structure, chemical-specific, steady-state venous blood concentrations were estimated across chemicals associated with unit oral and inhalation exposure rates and with chronic exposure at the identified exposure reference values. The thus-derived screening values—estimates of average blood concentrations—were then consistent with what would be expected in a typical adult human exposed at steady-state to the identified reference values. These screening values might be rough. But they do allow comparison of measured blood VOC concentrations to a benchmark consistent with existing risk assessments for these compounds rather than bright lines separating safe from unsafe exposure levels. Such a comparison can assist in the integration of these biomonitoring data into risk assessment, management, and prioritization decisions. Thus, in this instance, fully developed PBPK models, while useful, are not required [63, 71]. The BE values can be used as screening levels and can also be used to classify chemicals into low, medium, and high-priority categories for risk assessment followup.

## 9. Conclusions

After decades of toxicity testing, the emerging reality is that routine toxicity testing cannot fill the large data gaps that daily confront data generators (experimental scientists) and data users (assessors/regulators). And recent years have seen a shift away from studying health effects in whole animals. This shift also serves to refine, reduce, and replace animal use as the basis of ICCVAM legislation. In fact, more recently, this shift has spurred the use of human *in vitro* systems and high-throughput data generation.

As new chemicals and contaminants enter the environment, reliance on in silico modeling will increase. PBPK, PBPK/PD, BBDR models are tools that will help establish the cause-effect relationship—the basic tenant of risk assessment. The chief advantage of these tools, particularly the PBPK models, is their predictive power. It is this power that fills database gaps through simulations based on meticulously articulated scientific facts.

Many scientifically accurate and advanced PBPK models have been developed to evaluate carcinogenic and noncarcinogenic health effects. They are capable of route-to-route, exposure duration, interspecies, and other extrapolations commonly used in risk assessment. But health risk assessors—most of whom have limited experience with simulation software—are uncomfortable with the multiple simulation languages such as MatLab, Simusolve, and AcslX used to code those simulations. This limitation also restricts field application of the models in public health practice. Even experienced PBPK modelers, due to the lack of key information or equations, sometimes face problems when reconstructing published PBPK models for application. For these reasons, risk managers, decision makers, and the risk assessment community have been hesitant to adopt them. Having the models in easier-to-use form capable of solving real life problems will greatly increase their value and will help integrate technological and scientific advances into decision making.

PBPK models can also be useful in targeted research. They can estimate target organ concentrations of chemicals and integrate such information to predict whole-animal exposures. Because they are designed to determine the internal tissue concentration of a chemical from multiple exposure routes, they can help to determine the appropriate dose in target organs that could be used in *in vitro* toxicity testing systems. Combining *in vitro* pharmacokinetics and pharmacodynamics information could produce a concentration suitable for risk assessment [73, 74]. Once identified, the *in vivo* exposure corresponding to the *in vitro* concentration could be estimated through *in vitro-in vivo* extrapolation [75, 76]. Developing appropriate dose metrics, incorporating mode of action, and other chemical specific information could predict *in vivo* dose response curves from *in vitro* data [66, 76, 77]. But such extrapolations as determination of human external exposures to VOCs based on measured blood levels need careful assessment of the interval between exposure and sampling time since the latter only represent the concentration at the sample time and are a product of complex exposures from multiple routes and multiple sources [66, 78–80].

Hypothetically, reverse dosimetry shows the opposite could also be true [62, 64, 66]. That is, if we know the *in vitro* concentration that causes adverse effect(s) at a cellular or organ level, we could use these models to extrapolate to *in vivo* tissue level and ultimately to a human-allowable external dose. Some advances are being made towards this goal by integrating human dosimetry insights gained through *in vitro* studies and high throughput screening [81, 82]. Employing computational techniques and new simulation platforms, it has been shown that estimated oral equivalents could then be compared with allowable human exposures through environmental media.

In summary, though these models have not yet realized their potential, in silico toxicology is a growing field that will produce new and innovative computational tools. High-throughput screening and *in vitro* testing are changing toxicology testing strategies. In fact, such tests are helping to create the next generation of computational tools. But development of in silico tools should continually consider the two critical qualities necessary for the end-user—ease of use and accessibility. Training, increased transparency, and enhanced application could also help in-silico tools' acceptance as a real-life decision making tool. Finally, translational research, such as the development of the human PBPK toolkit, could further contribute to in-silico tools' accessibility and ease of use.
